# Ten simple rules in biomedical engineering to improve healthcare equity

**DOI:** 10.1371/journal.pcbi.1010525

**Published:** 2022-10-13

**Authors:** Olivia L. Lanier, Mykel D. Green, Gilda A. Barabino, Elizabeth Cosgriff-Hernandez

**Affiliations:** 1 Department of Biomedical Engineering, University of Texas at Austin, Austin, Texas, United States of America; 2 Olin College of Engineering, Needham, Massachusetts, United States of America; Dassault Systemes BIOVIA, UNITED STATES

## Introduction

In an address at the Convention of the Medical Committee for Human Rights in 1966, Dr. Martin Luther King Jr. stated, “Of all the forms of inequality, injustice in healthcare is the most shocking and inhumane [1].” Despite this call to action, there remains a great divide in health outcomes today with statistics that are staggering and unjust. For example, babies born to black women in the United States die at more than double the rate of babies born to white women [2]; black patients have higher rates of mortality than white patients from many diseases, including inflammatory bowel diseases and cancer [3,4]; American Indians and Alaska Native populations experience increased rates of cardiovascular disease and related risk factors [5]. Women, especially black women, experience higher rates of myocardial infarction or fatal coronary heart disease [6,7]. Each of these instances illustrates the prevalence of health disparities in diseases, with racial and ethnic minority patients being 1.5 to 2 times more likely than white patients to have major chronic diseases [8]. Health disparities have been categorized across race and ethnicity, gender, sexual identity and orientation, disability status or special healthcare needs, and geographic location (rural and urban). The unprecedented nature of the Coronavirus Disease 2019 (COVID-19) pandemic has brought these disparities into the spotlight and reignited the conversation about how to improve health equity in our country.

**Fig 1:**
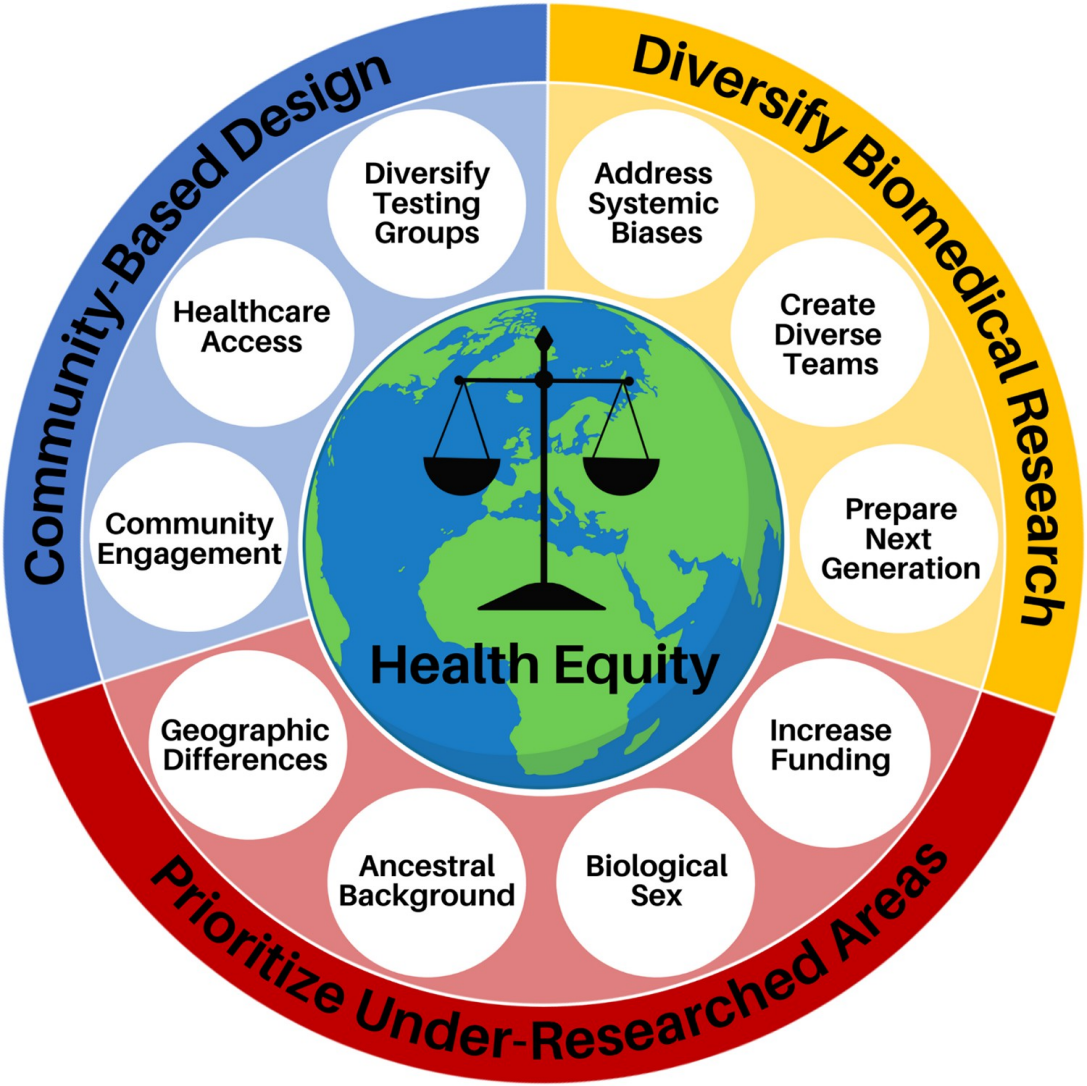
Overview of 10 simple rules in biomedical engineering to improve healthcare equity divided into 3 categories: (i) improving diversity and equity in biomedical research; (ii) increasing research of under investigated areas; and (iii) considering diverse communities in the research design process. Image created with BioRender.com.

**Fig 2:**
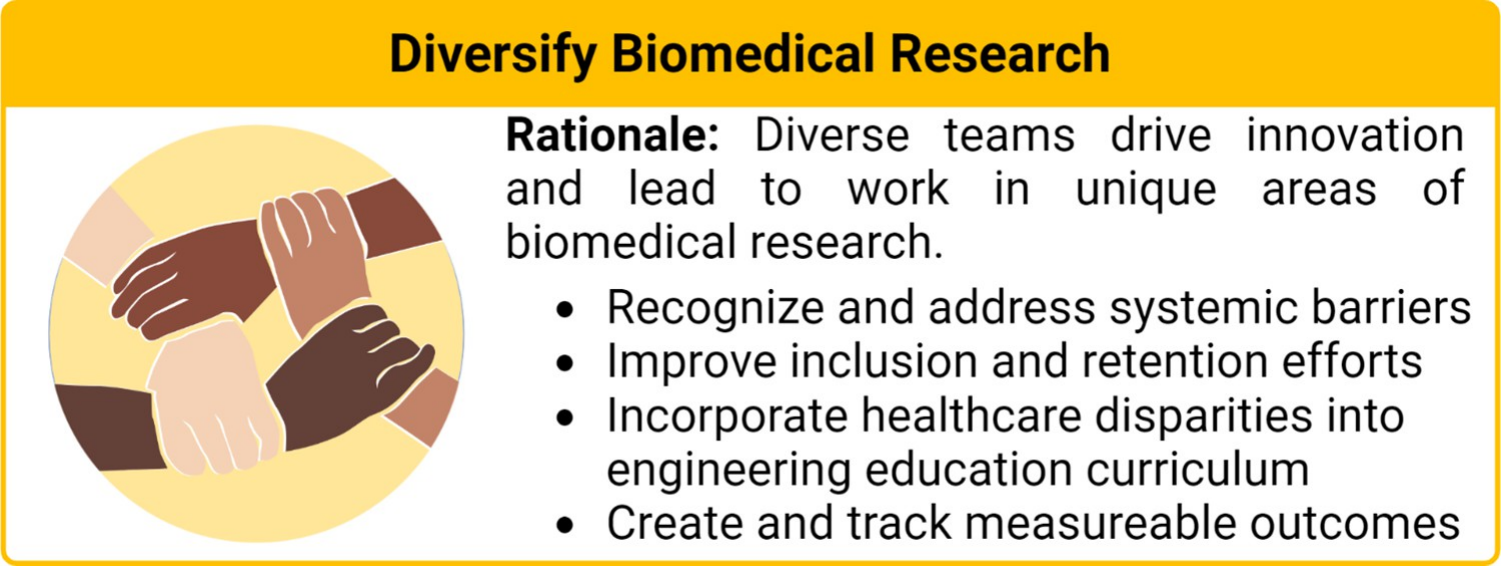
Rationale for diversifying biomedical research and takeaways from Rules 1 through 3. Image created with BioRender.com.

**Fig 3:**
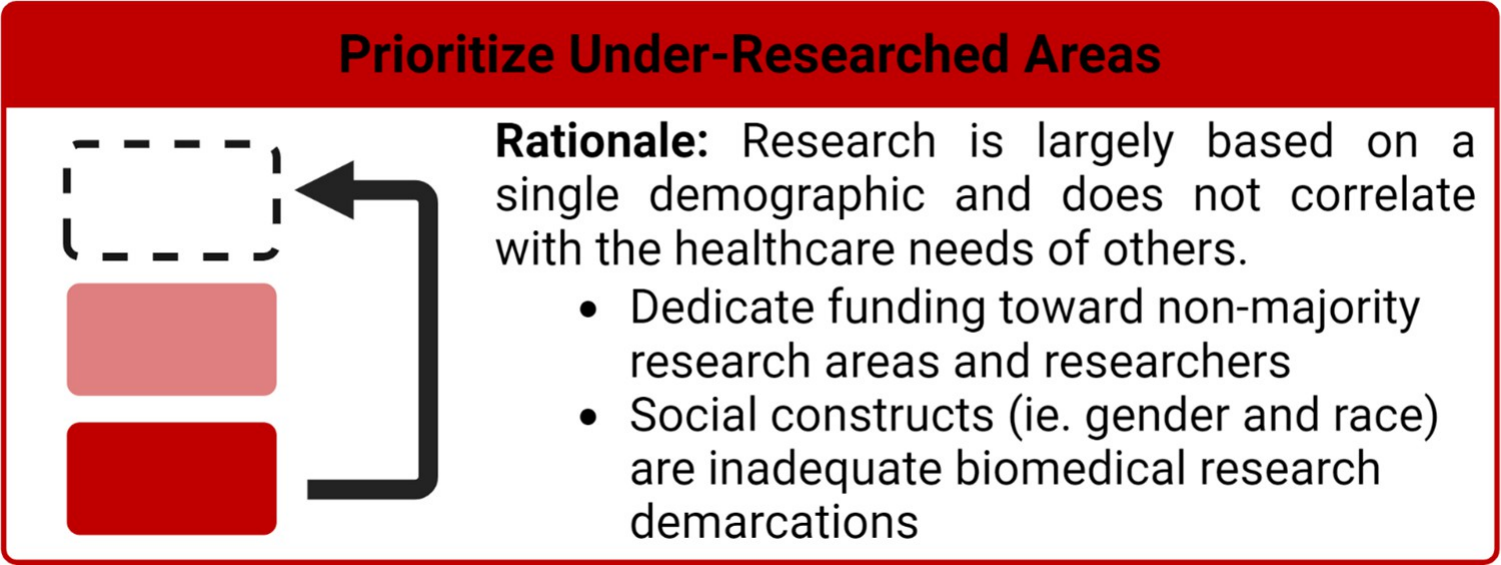
Rationale for prioritizing research in under investigated areas of healthcare disparities and diversifying biomedical research and takeaways from Rules 4 through 7.

**Fig 4:**
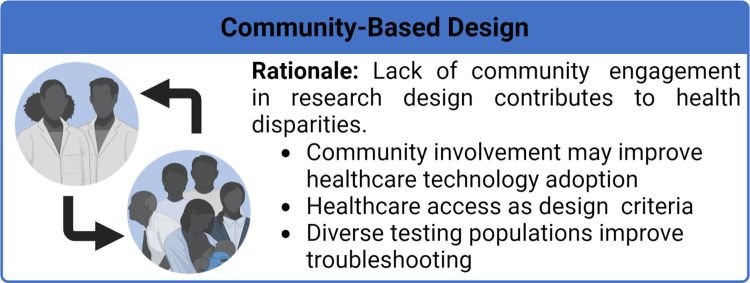
Rationale for prioritizing community-based design and takeaways from Rules 8 through 10. Image created with BioRender.com.

Health disparities are defined as preventable population-specific differences in the burden of disease, health outcomes, or access to healthcare [9]. Here we focus on healthcare disparities, which refers to differential access, use, and quality of medical care. Social determinants and implicit bias are well established as drivers of health disparities [10]; however, the impact of biomedical engineers who develop healthcare technologies that further propagate these inequities has only been implicitly stated. Given that nondiverse research teams have predominantly led medical device and therapeutic research, it is not surprising that the individual needs of different communities are often not considered in the design and optimization processes. This has resulted in numerous cases of technologies and therapies, be it unknowingly or not, that render the technology either ineffective or hazardous, in particular for women and racially minoritized populations. For example, pulse oximeters, which are used to monitor a patient’s supplemental oxygen needs and guide diagnostic decisions, were found to be 3 times less likely to detect hypoxemia in black patients as compared to white patients [11]. Furthermore, therapeutic dosing has been historically only determined in men whose metabolism is generally faster than that of women, leaving women at a higher risk [12,13], and in regard to biomaterial design, researchers have previously not considered differences in skeletal structure between men and women [14]. Other illustrative examples of these harmful oversights are discussed throughout this manuscript. As biomedical engineers developing the next generation of healthcare technologies, we are poised to either improve the health disparity landscape or further widen the gap. In this article, we provide researchers with 10 simple rules in biomedical engineering to improve healthcare equity. As shown in **Fig 1**, these rules revolve around 3 principles: (i) improving diversity and equity in science, technology, engineering, and mathematics (STEM) (Fig 2); (ii) increasing research on underserved areas and populations (Fig 3); and (iii) considering diverse communities in your research design process (Fig 4).

### Improving diversity and equity in STEM

#### Rule 1: Recognize systemic biases and track health disparities

Systemic biases, including structural racism, sexism, ableism, ageism, and other biases based on sexual orientation, gender identity, and socioeconomic status, contribute substantially to health inequities [15–17]. As a first step to rectifying these issues, we need to collectively recognize their existence as a society and actively work to eliminate them. These inequities go beyond discrimination from individuals into societal structures that systemically disadvantage members from historically excluded groups [15,18,19]. Historically, racism has played a critical role in creating the US, from exploiting land from Indigenous Peoples and Mexicans, bringing Africans in shackles for slavery, and inhumane treatment of Chinese immigrants as laborers [20]. “Color blind” initiatives fail to address racial inequalities infused in this country’s structural foundation. On an individual basis, implicit bias feeds systemic racism [21–24]; therefore, we need to dismantle our implicit biases by actively reframing negative associations, seeking out opportunities to educate ourselves, and having positive interactions/contact. Systemic racism has seeped into the academic world and marginalizes researchers from historically excluded groups [25]. In solidarity, over 10,000 academic faculty signed the pledge acknowledging systemic racism in academia [26]. We encourage our readers to also stand in solidarity to acknowledge and combat these structural inequities going forward.

In order to obtain equitable healthcare for all, we first need a better way to identify healthcare disparities and define the problems to be addressed. To this end, a system to evaluate equity in healthcare technology should be developed to establish a baseline for evaluation and tracking of health disparities across demographics with universalized definitions and standards [27]. The National Cancer Institute (NCI) recently created an Equity and Inclusion program, which includes systemic tracking and evaluation of equity activities [28], with an equity council to set criteria for assessing the progress of NCI’s equity efforts through measurable outcomes. This NCI equity council should be used as a model to create a broader council to do similar work on healthcare technology. Measurable outcomes could include the disease statistics and outcomes across demographics, the number of fatalities in minority versus majority populations, how many patients with the afflicted disease can afford the technology available, number of proposals written and funded on underserved diseases and conditions, and demographic representation in clinical trial testing. The council should also include diverse groups of people and reevaluate its objectives and findings at regular time intervals.

#### Rule 2: Promote diversity in teams and create inclusive environments

The scientific workforce does not reflect the societal demographics of the US, with women composing only 28.4% and black and Hispanic scientists accounting for only 5.4% and 6%, respectively [29]. Increasing the diversity in teams has been a recent focus in many fields, frequently coupled with the idea that diversity drives innovation [30]. In healthcare, diverse teams that reflect the patient population have additional potential benefits in improving patient outcomes [31]. The individual identity and personal experiences of a researcher often informs their area of interests and expertise [32]. For example, the emergence of women into biomedical research has significantly increased the number of studies on women’s health since the late 1970s [33,34]. Currently, the scientific work does not adequately address the healthcare needs of several communities [32]. By increasing the perspectives and interests on research teams, we can help ensure that the healthcare needs of every community are addressed.

To improve diversity and success of engineering teams, initiatives have focused on intentional recruiting and creating an inclusive climate where every team member is valued. To increase the diversity of the scientific pipeline, efforts have focused on increasing interest in healthcare research as a profession through local K-12 community outreach efforts hosted by the institution or recruiting at specialized events such as the Annual Biomedical Research Conference for Minority Students. Other efforts include collaborations with Minority Serving Institutions where students physically attend both institutions, such as the Atlanta University Center Consortium Dual-Degree Program that facilitates the enrollment of Historically Black College and University liberal arts students in 1 of 9 traditional engineering institutions. Enrichment programs, such as the Meyerhoff Scholars Program at the University of Maryland, Baltimore County, are known catalysts for promoting student success through financial scholarship, sense of belonging to a community, development of science identity, and preparation for professional STEM careers [35,36]. Although these programs are largely considered successful in increasing the number of scientists from historically excluded groups, they typically exist in select institutions or are not sustained due to lack of financial support [37]. It is important not only to increase diversity in your team, but also to focus on retention and ensure that historically excluded scientists have the required resources and support to thrive. Professional societies such as National Society of Black Engineers, Latinos in Science and Engineering, Society of Hispanic Professional Engineers, and the Society for Women Engineers serve as safe spaces that combat feelings of exclusion and discrimination. Beyond supportive communities, it is also important to educate all team members on diversity and inclusion issues to create a culturally competent scientific community.

#### Rule 3: Prepare the current and next-generation engineers to address health disparities

Substantial effort is needed to change how medical care providers and researchers are trained and educated. We should approach engineering education from a humanistic standpoint and promote awareness and recognition of systemic biases and health inequities while maintaining scientific rigor. Service-learning has been used to expose students to health disparities in the community with guided reflection [38]. This method also allowed the campus to grow and maintain a connection with community populations that experience health disparities and provided educational experiences to these populations. Education on various topics related to diverse community needs can also be explored and incorporated into the standard curriculum, including public health, health inequities, historical trauma, women’s health, LGBTQ+ issues, and cultural differences. For example, a previous case study discussed the integration of public health topics of health disparities and social determinants of health into a controlled drug delivery course [39]. This helped engage students in inclusive practices and highlight awareness of health disparities as consideration for engineering design. Moreover, supplemental education can be provided through showcasing health inequity researchers at conferences and professional meetings, hosting journal clubs that highlight research and accomplishments of underrepresented scientists, and highlighting books that educate about health inequities.

### Increasing research on underserved areas

#### Rule 4: Prioritize research and increase research funding on underserved diseases and populations

Many diseases that disproportionately affect historically excluded groups are often neglected in biomedical research with an associated lag in medical advances to improve care. Delayed diagnosis and lack of interventional care are common causes of death or disability for these patients. Outside of the US, tropical diseases (e.g., dengue, lymphatic filariasis, trachoma, leishmaniasis) are a neglected area of research that afflicts more than 1 billion people who live in low socioeconomic conditions across the world [40]. Many biomedical engineers working in oversaturated fields can apply their expertise toward these underserved diseases and make a real difference. To increase research in these areas, we must also look at the funding structure. Less prevalent diseases are typically underinvestigated due to the lack of sufficient funding. However, this is not the case for all conditions. Cystic fibrosis, which is mainly associated with white Americans, receives 3 times more funding per patient than sickle cell disease, a disease stereotypically associated with black people [41]. These discrepancies highlight that diseases that disproportionately affect historically excluded populations are not equitably financed and the associated decrease in research productivity hinders novel therapeutic development.

We previously noted in Rule 2 that a researcher’s identity affects their research interests. Thus, the diversity of the researchers will affect the level of effort to address health disparities. Although the National Institutes of Health (NIH) actively emphasizes the need to decrease health disparities, underrepresented investigators (black scientists in particular) are approximately 55% less likely to be funded than white authors of similar academic stature [42,43]. This lower funding rate has a strong effect on tenure and promotion rates that ultimately dictates the diversity within the discipline [44]. Some proposed solutions to address this inequity include altering the grant review process to ensure that all submissions are adequately discussed by the review panel and the consideration of the research team diversity when scoring the “Investigator” section. Private sector and philanthropic funding agencies are another potential source of funding that are sometimes dedicated to the advancement of global health and mitigating health disparities (**Table 1**). However, many of the private funding sources use the number of NIH grants already received to measure an application’s strength; this mechanism of funding may be better suited for experienced researchers to journey into health disparities. Collectively, there is an urgent need to increase funding for underserved diseases that disproportionately affect historically excluded populations and prioritize funding for diverse teams to increase the focus on addressing health disparities.

**Table 1:** Annual expenditure and biomedical research areas of interests for US-based philanthropic funding agencies. Table adapted from [45].

Cancer (Annual Expenditure, USD Millions)		Multidisciplinary + Global Health	
American Cancer Society	162.5	Howard Hughes Medical Institute	752.6
The Leukemia & Lymphoma Society	74	Bill & Melinda Gates Foundation	462.6
Susan G. Komen	58.9	World Health Organization (WHO)	133.6
The Breast Cancer Research Foundation	45.7	The Ellison Medical Foundation	47.1
St. Baldric’s Foundation	25.6	Simons Foundation	42.5
American Association for Cancer Research Foundation	23.7	Burroughs Wellcome Fund	31.3
Multiple Myeloma Research Foundation	14.5	The Medical Foundation	18.3
The V Foundation for Cancer Research	13.8	Doris Duke Charitable Foundation	16.4
Cancer Research Institute	13	Cures Within Reach	0.7
Damon Runyon Cancer Research Foundation	12.5	Flinn Foundation	3.9
Melanoma Research Alliance	8.4	Rockefeller foundation	UND
Conquer Cancer Foundation	7.6	The Donaghue Foundation	1.1
Children’s Tumor Foundation	6.4	The Pew Biomedical Programs	UND
Lymphoma Research Foundation	3.1		
LUNGevity Foundation	2.5	**Cardiovascular**	
American Brain Tumor Association	2.1	American Heart Association	135.6
Pershing Square Sohn Cancer Research Alliance (PSSCRA)	1.2	Doris Duke Charitable Foundation	16.4
MPN Research Foundation	0.4		
Rita Allen Foundation	UND	**Diabetes**	
		JDRF	111.7
**Neuroscience**		American Diabetes Association	35.8
Alzheimer’s Drug Discovery Foundation	65	Iacocca Family Foundation	1.8
Alzheimer’s Association	28.4	The Leona M. and Harry B. Helmsley Charitable Trust	UND
Autism Speaks	24.6	The Donaghue Foundation	1.1
Foundation Fighting Blindness	17.8	Lupus Foundation of America, Inc.	1
BrightFocus Foundation	10		
Parkinson’s Disease Foundation	5.1	**Pediatric, Aging, Women’s**	
CURE (Citizens United for Research in Epilepsy)	2.8	March of Dimes	18.7
Hydrocephalus Association	0.2	The Gerber Foundation	2.8
The Kavli Foundation	UND	The New York Stem Cell Foundation	4.8
Rita Allen Foundation	UND	Avon Foundation for Women	15.2
		American Federation for Aging Research	7.3
**Muscloskeletal**		The Donaghue Foundation	1.1
National Multiple Sclerosis Society	48.5	St. Baldrick’s Foundation	25.6
Rheumatology Research Foundation	10.8		
Arthritis Foundation	9.4		
Parent Project Muscular Dystrophy	2.1	UND, Undetermined	

#### Rule 5: Research sex-based determinants of health

Women suffer from a multiplicity of health disparities, ranging from higher incidences and mortality rates of numerous diseases to suffering from increased side effects from therapeutics [46]. These disparities can arise from various factors, including gender-based and sex-based differences in health and healthcare. Gender is a social construct that affects people’s perceptions, interactions, power, and resources and contributes to disparities through sociocultural factors such as diagnosis and treatment bias, psychological stressors, exposure to violence, and physical activity levels [47]. Sex is a biological factor dependent on genes, sex steroid hormones, and reproductive organs and is an important consideration for biomedical researchers involved in the design of healthcare technologies [48]. Recent research has revealed sex-based differences in immune system responses [49], microbiome composition [50], presentation of multiple diseases and conditions [51], therapeutic dosing and metabolism [12,52–54], and nanotechnology biodistribution and effectiveness [55,56]. Sex-based differences have been hypothesized to be due to differences in the X and Y chromosome as the X chromosome encodes greater than 1,100 genes compared to only approximately 100 genes encoded by the Y chromosome, X inactivation where female cells inactivate one X chromosome in each cell leading to a mosaic pattern that is not found in males, differences in levels of sex steroid hormones, and differences in reproductive organs [49,57]. Moreover, differences in pharmacokinetics and pharmacodynamics have been attributed to differences in fat and body water content, steroidal sex hormone levels, genetic phenotype, expression of hepatic drug-metabolizing enzymes, and more differences in absorption, distribution, metabolism, and elimination due to differences in bodily functions [12,52–54]. These differences can cause important sex-based differences in performance that should be addressed by engineers. For example, they can affect the efficacy of nanotechnologies [55,56] or the acceptance of biomedical devices within the body [58]. Differences in skeletal structure, bone strength, and properties of muscles, tendons, and ligaments also make sex an important variable to consider for implant and biomaterial design [59]. Unfortunately, it has been shown that most research does not analyze results with sex as a scientific variable unless the topic is reproduction [12]. It is important to recognize that sex-based differences are not binary and occur on a spectrum that can be influenced from a person’s age, gender/sex identity, environment, and more. In particular, the LGBTQ+ community faces a plethora of health disparities [60].

In biomedical research, there is often a “one-size-fits-all” approach that has been predominately driven by male determinants, disproportionately leaving women at a higher risk. We need to better understand these differences to create inclusive designs that benefit everyone. To help researchers understand these differences, funding agencies have begun to fund this research. The NIH is applying processes to help balance sex in cell and animal studies and has created initiatives for women’s health issues [61]. The Food and Drug Administration (FDA) urges researchers to account for sex and gender differences in drug metabolism [52]. Furthermore, there are foundations such as the Foundation for Gender-Specific Medicine investigating how biological sex and gender affect human function and disease [62]. We encourage researchers to consider sex and gender appropriately as research variables when developing new healthcare technologies, as this could lead to the discovery of new mechanisms of disease in addition to promoting more equitable healthcare technologies.

#### Rule 6: Research ancestral biological determinants of health

Race is another social construct that has led to the development of numerous injustices and inequities within and outside the context of health. Although the stratification of populations by race facilitated the initial identification of health disparities, this context is more closely linked to the social determinants of health rather than biological determinants [63]. The way race has developed as a concept is more related to how a person looks and is treated by society rather than genetic ancestry. For example, people of mixed African and European descent, such as Barack Obama, are often considered black, demonstrating that ancestral background does not necessarily correspond to a person’s perceived race [64]. Race groups individual populations whose ancestral origins have varying cultural and biological differences; case in point, people from China and India are classified as “Asian” in western society [65]. Furthermore, using race as a health indicator is harmful and viewing a patient only from the context of “race” does not factor in the genetic admixture of the modern world and the allele frequencies that accompany it. This was the situation for a black child who suffered chronic lung complications that remained undiagnosed with cystic fibrosis for years until their X-rays were examined without knowing the child’s race [66]. Many clinical decision-making tools also use inappropriate race correction factors that lead to worsened health outcomes for racially minoritized patients [67].

Evidence suggests ancestral genealogical origins are a more reliable and inclusive tool to study population-level health tendencies [68]. For example, Latino Americans are the most prominent and fastest-growing historically marginalized population in the US, whose ancestral genetics vary. Mexican Americans comprise Native American and European ancestry; however, Puerto Ricans have a more prevalent European and African mixture [69]. Ancestry has been shown to affect biological variables between people; for example, people of sub-Saharan African descent have been shown to have a stiffer sclera than people of European or mixed descent [70]. Ancestry can also affect disease frequency; thalassemia is a genetic blood disorder whose symptoms manifest from mild to severe based on which variant of the disease the patient inherits and is common in people from specific regions of the world [71]. Therefore, genetic testing is the simplest solution to identifying genetic disease and traits of an individual that may otherwise remain unknown. As biomedical engineers, understanding this concept is critical to research design because the genetic background of cells has been recognized as a source of variability in tissue-engineered constructs [72] and influences drug pharmacokinetics and pharmacodynamics [73]. However, recent insights into the ethnic makeup of commercial cell lines demonstrated that reporting origin is not common practice, and from those that were reported, nearly 55% of the cell lines came from white donors and an additional 25% are from unidentified donors, further perpetuating the homogeneity of biomedical research [74]. This is an area that needs more rigorous study to identify key determinants in biomedical research to better design medical devices and therapeutics that serve all communities.

#### Rule 7: Research geographical and environmental determinants of health

The unique environmental exposures of where one lives can also profoundly affect their overall health. Environmental factors can vary between the national scale (e.g., first and developing countries) and local (e.g., Flint, Michigan water crisis). External and internal stressors such as pollution [75], psychosocial stress [76,77], and traumatic experiences [78–80] have been associated with disease development through the epigenetic modification of the genome. Air pollution has also been implicated in adverse effects on maternal and perinatal health [81], an area associated with significant disparities as black mothers are almost 3 times more likely to die during labor than non-Hispanic white mothers [82].

Recent work has also revealed geographic differences of the population concerning global health. For example, global disease patterns may be associated with local gut microbiota composition, through which transplantation of more robust microbiota strains transfer immune resilience [83]. The microbiome and nutrition status has also been shown to affect the development and functioning of the immune system differently in males and females, showing that environment and sex should be considered intersectional variables [49]. Moreover, environment and genetic ancestry could play an intersectional role in disease development. As engineers, we can develop model systems to gain insight into these unique conditions and devise strategies to address these differences. Furthermore, developing more geographically strategic biomedical engineering facilities may help address local healthcare technology disparities.

### Considering diverse communities in your research design process

#### Rule 8: Make designs that promote diverse community adoption

When designing a new therapy or medical device, we need to involve the community from the inception of the idea to final adoption. We can gauge acceptance of therapies and improve patient compliance by modifying our technologies to the patient’s needs and lifestyles. Complex treatment regimens, which can include frequent visits to healthcare professionals for infusions or laboratory tests, strict medication timetables, self-monitoring, and upkeep of specialized medical equipment, can lead to reduced patient compliance and are particularly burdensome on a patient’s ability to work and maintain a certain lifestyle [84]. We need to design realistic treatments and take into account the treatment’s route, frequency, and complexity. The introduction of community-based participatory research (CBPR) has challenged how scientists should conduct research by encouraging a systematic effort to develop relationships between biomedical researchers and the community.

CBPR can also be used to address adoption challenges related to institutional distrust, misinformation, and cultural differences. These challenges have existed for centuries as a result of historical trauma in the medical field. One of the most egregious acts of unethical human experimentation comes from the gynecological work of J. Marion Sims, who performed countless experimental surgeries on enslaved women in the 1840s, most of which were unanesthetized due to the inaccurate belief of blacks having a higher pain tolerance [85]. Other notorious acts include the Tuskegee Syphilis Experiment [86], the forced sterilization of the Indigenous women [87], and the dermatology study on inmates at Holmesburg Prison [88], all of which were carried out after the World War II drafting of the Nuremberg Code. Although the development of the Belmont Report in 1979 strengthened ethical guidelines of scientific research through informed consent, distrust of the US Healthcare System and Clinical Research varies between 20% to 80% depending on age, race, ethnicity, and socioeconomic status [89–91]. Engaging the community as collaborators rather than subjects may help rectify the public distrust of biomedical research and identify cultural barriers to adoption earlier in the design process. In addition, having diverse team members can help further build trust with patients and improve communication with historically marginalized or abused communities.

Finally, linguistic barriers can also hinder community adoption and worsen health outcomes. It has been shown that how we disseminate new healthcare advances to populations with limited English proficiency and low literacy is associated with patient perception of the technology [92]. It is the lack of appropriately tailoring educational materials to the targeted demographic that promotes community apprehension and deters initiation of beneficial healthcare technologies such as vaccines [93]. We must be cognizant of interpretation errors when explaining our healthcare technologies to all stakeholders including clinicians, investors, politicians, and patients. Implementing CBPR in biomedical engineering has the potential to specifically address health disparities through the incorporation of genuine conversations between academics and underserved patient populations that promote mutual learning, sharing of resources and knowledge, and serve as a force for social change [94].

#### Rule 9: Consider healthcare access in biomedical design

Biomedical engineers should also consider healthcare access in their initial design. People of low socioeconomic status or in rural areas in the US have less access to healthcare technologies due to cost or proximity to medical facilities. The cost of therapy or medical devices has long contributed to health disparities across socioeconomic demographics. This is only partially mitigated by health insurance, given the lack of universal health coverage and reimbursement differences. Beyond financial considerations, there are issues related to access due to travel restrictions and service availability. Limited transportation or sick leave can substantially restrict what therapies and services a patient may receive. For example, the American College of Obstetricians and Gynecologists reported in 2014 that less than one-half of rural women live within a 30-minute drive to the nearest hospital offering perinatal services. These distances to healthcare facilities become further for rural areas with high black or Indigenous populations [95]. Moreover, technologies that require cold storage, special equipment, or high amounts of electricity to run are not accessible. The need for cold storage for COVID-19 vaccines was substantially limited where the vaccines could be administered and created differential access across communities. Therefore, engineers should consider the required storage conditions of their therapeutics and technologies during the design phase; numerous efforts are being made to improve the cold chain manufacturing dilemma by engineering a more insulated carrier box or the design of vaccine formulations that are stable at high temperatures for months that circumvents this issue altogether [96,97].

Telemedicine, which gained mass popularity in conjunction with the pandemic in the US, can address some of these regional access restrictions but has implementation challenges for the nearly 25 million people with low digital literacy, limited English proficiency, and financial disparities [98,99]. Point-of-care technology has also proven to be a vital area for addressing healthcare access disparities. In the US, smartphone applications and accessories are abundant dedicated to monitoring, tracking, and improving an individual’s health. The introduction of smartphones and the internet to India’s rural population has positioned the country as one of the fastest-growing technology markets. Coincidentally, nearly 20% of the global burden of disease originates from the 39 million impoverished people in India [100]. Smartphones are becoming a ubiquitous technology in a country where 70% of the population resides in rural areas [101]. Research groups such as the Richards-Kortum lab have pioneered the development of numerous screening and diagnostic technologies specifically tailored to the needs of developing countries [102]. They advise to design simple solutions, not to overlook traditional solutions, think long term while solving short term, engage students in frugal design, and design for context [103]. An example of frugal design is the introduction of paper-based microfluidic platforms, which have provided cost-effective analytical devices with a wide range of applications, ease of fabrication/operation, and equipment independence [104]. Another example that engaged students in frugal design is when students work together to design a cheap and simple neonatal monitoring device [105]. Successful adaptation of this technology to address the population’s healthcare needs can affect the patient–physician relationships, improve patient buy-in, and overall healthcare system efficiency. As biomedical engineers, we must consider the cost, supply chain, storage conditions, and regional access of the technologies we are developing and whether this may create a scenario with differential patient access.

#### Rule 10: Evaluate diverse testing populations in experiments

In addition to inclusive design and community adoption considerations, we need to ensure that new medical therapies are tested in a way to ensure safety and efficacy for the full spectrum of the intended patient population. Reported scientific data are typically generated from more privileged populations, suggesting that our understanding of disease is inherently limited and biased [106]. For example, human genomic data play a critical role in biomedical research, but only 4% of the patients in the registry are of non-European ancestry [107]. The lack of diversity in testing groups has led to life-threatening complications in underserved populations, such as women overdosing on medication that was only tested in men [108]. Therefore, diverse groups should be represented throughout every step of the research design process for new healthcare technologies and therapeutics.

The NIH [61] and FDA [52] have created policies and guidelines to encourage the inclusion of women and historically marginalized populations in clinical trials as well as consideration of sex and age as biological variables in experimental design. In addition to increasing the diversity of cell origins and animal models, the development of modeling systems (in silico, in vitro, or in vivo) that account for the biological complexities of sex, ancestry, hormonal composition, and environmental/social factors can be used to further our understanding of disease from the cellular to whole-body scale [109,110]. Using modeling systems allows for diversity to be included earlier in the research process, but may also serve as a platform to bring attention to previously underserved areas such as transgender hormone therapy [111] or helping define the genetic ancestry of multiracial populations [112]. It is also our responsibility as future clinical trial sponsors to thoroughly design the preclinical testing models, so that we are able to broaden the enrollment eligibility criteria to ensure a diverse and inclusive trial [113]. We must work with insurance companies to cover our treatment to a wide range of patients to obtain the diversity a true trial needs. Additionally, we need to work to be effective science communicators to eliminate enrollment barriers due to the lack of trust in biomedical research in some communities that has arisen from historical trauma discussed previously.

Clinical trials are a prime area where CBPR may be immediately impactful. A recent NIH report indicates that racially minoritized populations represent approximately 30% of clinical trial enrollment, significantly increasing from nearly 10% during the mid-90s [114]. Unfortunately, during the emergency clinical trials for the COVID-19 vaccine, white individuals were overrepresented (78%) and American Indian, Alaska Native, Hawaiian, and Pacific Islander participation was not included in many studies (50% to 60%) [115]. This lack of representation is especially concerning given that racially minoritized groups have a higher vaccine hesitancy than whites but are more likely to contract the virus and be hospitalized with severe COVID-19 complications. Conscientious efforts to address community awareness and properly educate targeted groups about the science and safety of the vaccine are associated with reducing vaccine hesitancy [116–118] and may improve clinical trial enrollment [119].

## Conclusions

According to the preamble of the World Health Organization, health is a basic human right and it is our responsibility as healthcare researchers to help uphold this right for all people. We need to first acknowledge the existence of systemic biases and health disparities in our society and then work to eliminate them. In this work, we provided 10 rules to guide biomedical engineers and researchers to improve the health equity landscape (Fig 1). Many of these rules require large-scale, systemic changes including diversifying our technology workforce and balancing systemic funding disparities. This requires increased advocacy and sustained effort from our community of scientists and engineers to change the systems and practices that resulted in the current inequities and lack of diversity. In addition to these systemic changes, there are equity-minded practices in the engineering design process that every researcher can adopt today (e.g., sex-based differences, community adoption considerations). In the past, researchers may have been ignorant that differences across populations and communities can cause health disparities; however, there have been sufficient high-profile case studies to establish that we need to change our practices to consider these differences throughout the engineering design process and utilize diverse cohort testing [11,56,60,61]. As Maya Angelou said [120], “Do the best you can until you know better. Then when you know better, do better.” By approaching engineering design from a humanistic standpoint where we consider the needs of different communities, we can better work to advance healthcare outcomes for all members of our society.
